# Disturbed functional connectivity and topological properties of the frontal lobe in minimally conscious state based on resting-state fNIRS

**DOI:** 10.3389/fnins.2023.1118395

**Published:** 2023-02-10

**Authors:** Hui Chen, Guofu Miao, Sirui Wang, Jun Zheng, Xin Zhang, Junbin Lin, Chizi Hao, Hailong Huang, Ting Jiang, Yu Gong, Weijing Liao

**Affiliations:** Department of Rehabilitation Medicine, Zhongnan Hospital of Wuhan University, Wuhan, Hubei, China

**Keywords:** minimally conscious state, functional near-infrared spectroscopy, frontal lobe, functional connectivity, graph theory

## Abstract

**Background:**

Patients in minimally conscious state (MCS) exist measurable evidence of consciousness. The frontal lobe is a crucial part of the brain that encodes abstract information and is closely related to the conscious state. We hypothesized that the disturbance of the frontal functional network exists in MCS patients.

**Methods:**

We collected the resting-state functional near-infrared spectroscopy (fNIRS) data of fifteen MCS patients and sixteen age- and gender-matched healthy controls (HC). The Coma Recovery Scale-Revised (CRS-R) scale of MCS patients was also composed. The topology of the frontal functional network was analyzed in two groups.

**Results:**

Compared with HC, the MCS patients showed widely disrupted functional connectivity in the frontal lobe, especially in the frontopolar area and right dorsolateral prefrontal cortex. Moreover, the MCS patients displayed lower clustering coefficient, global efficiency, local efficiency, and higher characteristic path length. In addition, the nodal clustering coefficient and nodal local efficiency in the left frontopolar area and right dorsolateral prefrontal cortex were significantly reduced in MCS patients. Furthermore, the nodal clustering coefficient and nodal local efficiency in the right dorsolateral prefrontal cortex were positively correlated to auditory subscale scores.

**Conclusion:**

This study reveals that MCS patients’ frontal functional network is synergistically dysfunctional. And the balance between information separation and integration in the frontal lobe is broken, especially the local information transmission in the prefrontal cortex. These findings help us to understand the pathological mechanism of MCS patients better.

## 1. Introduction

Disorders of consciousness (DOC) caused by acquired severe brain injury from various causes are several states that include coma, unresponsive wakefulness syndrome (UWS), and minimally conscious state (MCS) (Owen, 2019). Prolonged DOC is defined as losing consciousness for more than 28 days (Kondziella et al., 2020). The survival time of patients with prolonged DOC is generally 2–5 years (Hirschberg and Giacino, 2011). It is called MCS when the patient appears to have any conscious perception of the surrounding environment with repetitive behavior, such as visual tracking or gazing at targets, pain localization, etc. (Giacino et al., 2002). Compared to UWS, patients in MCS have developed definite signs of consciousness and may have better aware potential (Song et al., 2020). The MCS may be the final state of consciousness in some DOC patients, or it may be a transitional state to further clear consciousness. Patients in MCS are bedridden for a long time and require specialized care, which increases the financial burden on the family (Owen, 2008). However, the neuropathological mechanism of MCS patients is still unclear.

As a relatively new imaging method, functional near-infrared spectroscopy (fNIRS) measures the changed concentration of oxyhemoglobin (HbO) and deoxyhemoglobin (HbR) in the cerebral cortex by emitting near-infrared light (Boas et al., 2014; Rupawala et al., 2018). Compared with functional magnetic resonance imaging (fMRI) and other functional neuroimaging technology, fNIRS is more portable, easier to wear, has lower detection cost, and is safer to scan in a natural environment for MCS patients in bed. Currently, fNIRS can monitor real-time physiological responses to quantify the stimulation parameters for different neuroregulatory techniques. Zhang et al. (2018) found increased local cerebral blood flow in the prefrontal cortex during spinal cord stimulation in patients with prolonged DOC. Moreover, some studies have monitored the residual consciousness of DOC patients while performing mental arithmetic (Kurz et al., 2018) and motor imagery tasks based on fNIRS (Molteni et al., 2013). However, judging whether a DOC patient completes the task paradigm is difficult because their attention and awareness may fluctuate over time (Abdalmalak et al., 2021). Resting-state fNIRS is measured when subjects are quiet, relaxed, and not performing specific cognitive tasks, which can reflect the cooperative and spontaneous activity between different brain regions. Compared with task state, resting-state data acquisition is easier to implement, and the results are more stable.

Functional connectivity refers to the temporal correlation of neuronal activity, and the strength of functional connectivity is measured by the correlation coefficient (Fingelkurts et al., 2005). Studies found that the functional connectivity strength in the resting-state brain networks decreased in patients with DOC compared with healthy people (Sinitsyn et al., 2018; Martinez et al., 2020). Another study found that the default mode network, frontoparietal network, sensorimotor network, and other resting-state functional networks of patients with DOC were extensively disrupted (Demertzi et al., 2015). Many studies consider the human brain as a complex brain network, and the brain network is a set of nodes and edges (functional connectivity between brain regions) (Bullmore and Sporns, 2009; Avena-Koenigsberger et al., 2017). The brain network often exhibits the balance of spontaneous integration and separation in function, which can be quantified by graph-theoretic topological analysis based on resting-state data (Bullmore and Sporns, 2012). Some studies found that the alterations of the brain’s network topology tended to occur in many disorders, such as depressive disorder (Zhang et al., 2011), Alzheimer’s disease (Stam et al., 2007), and post-stroke cognitive impairment (Miao et al., 2022).

To date, a few studies have used functional neuroimaging technology to explore the topological properties of patients with DOC and have identified changes in the frontal regions. Liu et al. (2023) found that the intraconnections within Brodmann area 10 and interhemispheric connections between Brodmann area 10 and Brodmann area 46 were helpful in distinguishing between MCS and UWS based on fNIRS study. One fMRI study found that the patients in MCS had increased nodal degree in the left superior frontal and decreased in the right orbital frontal (Crone et al., 2014). Another electroencephalography (EEG) study found that UWS patients showed decreased nodal degree and betweenness centrality in the frontal regions in β1 band (13–20 Hz) compared to MCS patients (Cacciola et al., 2019). The frontal lobe receives extensive neural projection connections from other cortical and subcortical regions. The complex pattern of fiber connections determines the functional complexity of the frontal lobe, a key brain region associated with many higher cognitive functions (Cusack et al., 2016). Several leading theories of consciousness suggest that the prefrontal cortex is inextricably linked to consciousness (Seth and Bayne, 2022), like higher-order theories (HOTs) (Cleeremans et al., 2020; Fleming, 2020), and global workspace theories (Dehaene and Changeux, 2011; Mashour et al., 2020). However, no studies have specifically explored the topological properties of the frontal lobe in MCS patients. We still do not know how the consciousness impairment caused by pathological injury affects the frontal network topology.

In this study, we hypothesized the disturbance in the functional connectivity and topology of the frontal lobe in MCS patients. We used complex network analysis to investigate the spontaneous integration and separation of information in the frontal lobe of MCS patients based on resting-state fNIRS data. Moreover, we explored the potential correlation between network topology that changed significantly between-group differences and the Coma Recovery Scale-Revised (CRS-R) scores.

## 2. Materials and methods

### 2.1. Participants

Sixteen patients in MCS and sixteen age- and gender-matched healthy controls (HC) were included in this study. Among them, sixteen HC were recruited from the community, and all the MCS patients were from the Department of Rehabilitation in Zhongnan Hospital of Wuhan University between November 2021 and June 2022. All patients were diagnosed as MCS based on continuously repeated (≥5) CRS-R assessed by a professional therapist (Giacino et al., 2004). The exclusion criteria were as follows, (1) The course of disease <28 days (Giacino et al., 2018); (2) Skull defect or after cranioplasty; and (3) History of psychiatric or neurological illness, such as Alzheimer’s disease, Parkinson or depression. This study was conducted in Zhongnan Hospital of Wuhan University, approved by the Medical Research Ethics Committee and Institutional Review Board of Zhongnan Hospital (2021126), and written informed consent was signed by the legal surrogate of each subject.

The CRS-R scale consists of six functional subscales addressing auditory, visual, motor, oro-motor, communication, and arousal level, totaling 23 hierarchically organized items (Giacino et al., 2004; Di et al., 2017). In addition to the total score, the single score of this scale was important to diagnose DOC patients.

The demographic and clinical data of all MCS patients are summarized in Table 1.

**TABLE 1:** Demographic and clinical data of MCS patients.

Index	Patient	Gender/age (years)	Etiology	Duration (months)	CRS-R
1	MCS-	F/49	Traumatic brain injury	7	10 (1/1/5/1/0/2)
2	MCS-	F/79	Intracerebral infarction	1.5	13 (1/3/5/2/0/2)
3	MCS-	M/31	Traumatic brain injury	12	11 (2/3/2/2/0/2)
4	MCS +	M/70	Intracerebral infarction	1	15 (3/1/5/3/1/2)
5	MCS +	M/53	Intracerebral hemorrhage	1	11 (3/1/5/1/0/1)
6	MCS-	F/85	Traumatic brain injury	1.5	9 (2/3/3/0/0/1)
7	MCS-	F/69	Intracerebral hemorrhage	1.5	8 (1/3/2/1/0/1)
8	MCS-	M/70	Intracerebral infarction	1	8 (2/3/2/1/0/1)
9	MCS-	M/53	Traumatic brain injury	11	12 (2/3/5/1/0/1)
10	MCS-	M/86	Traumatic brain injury	3.5	4 (0/0/3/0/0/1)
11	MCS-	F/82	Intracerebral hemorrhage	1	9 (1/1/3/2/0/2)
12	MCS-	M/65	Intracerebral hemorrhage	1	4 (0/0/3/0/0/1)
13	MCS-	F/54	Intracerebral hemorrhage	1	10 (1/3/5/0/0/1)
14	MCS-	M/83	Intracerebral infarction	3	11 (0/3/5/2/0/1)
15	MCS-	F/84	Intracerebral infarction	1.5	8 (0/3/2/2/0/1)

MCS, minimally conscious state; CRS-R, coma recovery scale-revised.

### 2.2. Data acquisition

**FIGURE 1:**
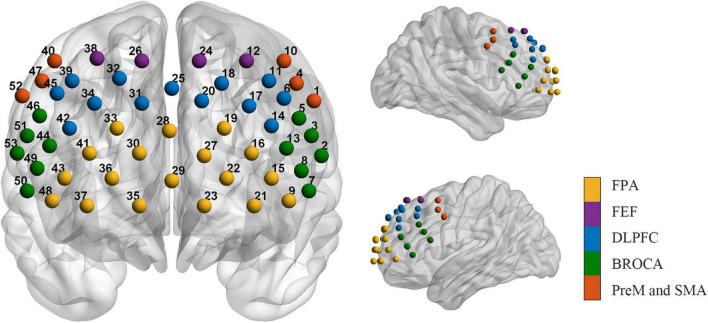
Arrangement of the 53 channels covering the frontal lobe. Including the PreM and SMA (red, Premotor, and supplementary motor area), FEF (purple, frontal eye fields), BROCA (green, Broca’s area), FPA (yellow, frontopolar area), and DLPFC (blue, dorsolateral prefrontal cortex).

The data were collected by a multichannel continuous wave near-infrared optical imaging system (BS-3000, Wuhan Znion Medical Technology Co., Wuhan, China). This system can emit two wavelengths of 690 and 830 nm at each source optic fiber with a sampling rate of 20 Hz and measure the changes in concentration of HbO, and HbR through optical attenuation. To normalize the fNIRS channels, we applied a 3D digitizer to record the exact spatial coordinates of 4 reference points (Nz, Cz, AL, and RL) and 32 probes (16 sources and 16 detectors with 3 cm source-detector-distance). Then the 53 channels were converted to an estimated Montreal Neurological Institute (MNI) space (Singh et al., 2005) by NIRS-SPM (Ye et al., 2009). Based on the Brodmann probabilistic atlas, all 53 channels were divided into the following five cortical regions: Premotor and supplementary motor area (PreM and SMA), frontal eye fields (FEF), Broca’s area, frontopolar area (FPA), and dorsolateral prefrontal cortex (DLPFC) (Figure 1). The lowest row of probes was aligned along the subject’s eyebrow arch, and the middle row of probes was parallel to the midsagittal line of the nasal root-occipital tuberosity. The subjects underwent a 5-min resting-state session of fNIRS measurement in a quiet evaluation room. They were required to keep still and relax the mind with their eyes opened (Yang and Hong, 2021).

### 2.3. Data pre-processing

Data processing was performed by Homer2 toolbox in the MATLAB environment (Huppert et al., 2009). Pre-processing procedures were as following: (1) Converting raw data to the optical density (OD) (Scholkmann and Wolf, 2013); (2) Trimming the first and the last 60 s; (3) Correcting motion artifacts by spline interpolation algorithm (Scholkmann et al., 2010); (4) 0.01–0.1 Hz bandpass filtering to minimize the physiological interference and produce the data with the best signal-to-noise ratio, such as blood pressure (Mayer) waves (∼0.1 Hz), respiration (∼0.4 Hz) and heart pulsation (1∼1.5 Hz) (White et al., 2009; Mesquita et al., 2010); and (5) Converting optical density to relative HbO/HbR concentration through the modified Beer-Lambert law.

Only HbO signals were analyzed in this study since they have a better signal-to-noise ratio than HBR and are more sensitive to monitoring regional cerebral blood flow (Fu et al., 2014).

### 2.4. Functional connectivity calculation and network construction

The three midline channels (channels 25, 28, and 29) were removed from the analysis because they did not belong to either side of the brain. The time series’ correlation coefficients (r) for each pair of nodes were calculated using Pearson correlation analysis. Fisher’s r-to-z transformation was used to normalize the correlation coefficients to *z*-values. The nodes were defined as channels, and the edges were defined as correlation coefficients between pairs of nodes. Thus, a 50 × 50 functional connectivity matrix was calculated for each subject. Our network analysis was confined to positive correlations. Negative correlation coefficients were set as zero because the biological explanation of negative correlations was ambiguous and equivocal in the correlation matrix (Murphy et al., 2009; Carbonell et al., 2014). Before using graph theory to quantify the network topology, we applied thresholds to retain only connections above a set threshold (0.4–0.9, 0.05 interval).

Since the 50 channels can be divided into ten regions of interest (ROIs) according to the Brodmann probabilistic atlas, we calculated the average nodal topological properties for all channels in each ROI, including the left PreM and SMA, left Broca’s area, left FEF, left FPA, left DLPFC, right PreM and SMA, right Broca’s area, right FEF, right FPA, right DLPFC.

### 2.5. Network analysis

The topological properties of functional network can be quantified by graph theoretic methods to reflect the functional integration and separation of the brain network, including global and nodal network metrics. All network metrics were computed in the GRETNA toolbox (Wang et al., 2015). We calculated several typical global topological properties: small-worldness (σ), clustering coefficient (Cp), characteristic path length (Lp), global efficiency (Eg), and local efficiency (Eloc). The node topological properties included nodal clustering coefficient (NCp) and nodal local efficiency (NLe). NCp refers to the likelihood that its neighbor nodes are also connected, and the Cp is defined as the average of the cluster coefficients of all nodes in the network. NLe refers to the communication efficiency between adjacent nodes after a node is removed, and the Eloc is the average of all NLe. Lp is the average of the shortest path lengths of any pair of nodes in the network. The Eg of a network is the inverse of the harmonic mean of the shortest path between any two nodes. The small-worldness of the network means that the network has a shorter Lp and greater Cp (Watts and Strogatz, 1998).

### 2.6. Statistical analysis

All statistical analyses were performed by SPSS 23.0. We used mean ± standard deviation to represent the numerical variables and Shapiro-Wilk (S-W) test to analyze the normal distribution. The chi-square test examined gender differences between groups. Two-sample *t*-tests were used to compare the group differences in age and the area under the curve (AUC) of global topological properties. Two-sample *t*-tests with false discovery rate (FDR) correction were performed for multiple comparisons in functional connectivity and the AUC of nodal topological properties (Storey, 2002). Pearson correlation analysis was performed between network metrics that changed significantly between-group differences and the CRS-R scale scores. *P* < 0.05 means a statistically significant difference.

## 3. Results

### 3.1. Demographic and clinical results

Fifteen MCS patients met the inclusion criteria, and one patient was excluded due to head movement. The clinical data of the recruited MCS patients are shown in Table 1. Fifteen MCS patients (eight males, age 67.53 ± 16.48 years) and sixteen HC (nine males, age 58.63 ± 14.51 years) were finally included in this study. There were no significant between-group differences in age and gender between HC and MCS groups (*p* > 0.05) (Table 2).

**TABLE 2:** Baseline of demographic and clinical data.

Characteristics	HC	MCS	*p*-value
Gender (male/female)	9/7	8/7	*p* > 0.05
Age (years)	58.63 ± 14.51	67.53 ± 16.48	*p* > 0.05
Duration (months)	–	3.23 ± 3.72	–
CRS-R scores	–	9.60 ± 2.95	–

HC, healthy controls; MCS, minimally conscious state; CRS-R, coma recovery scale-revised.

### 3.2. Between-group differences in functional connectivity

**FIGURE 2:**
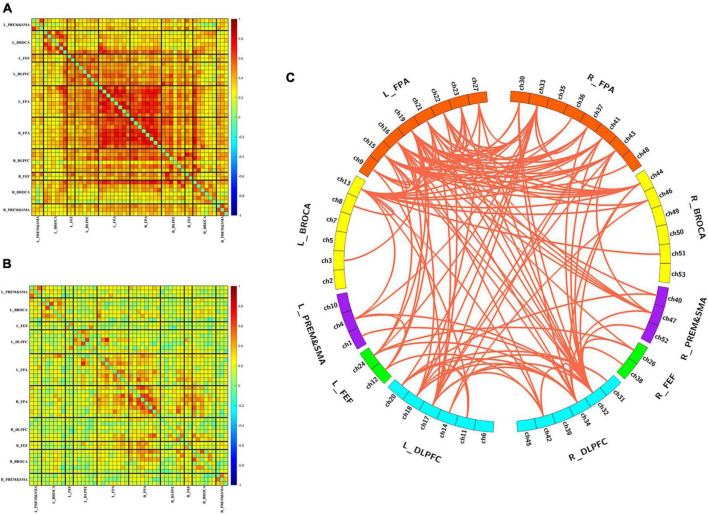
Differences in functional connectivity between HC and MCS groups. **(A)** Group-averaged functional connectivity metrics in HC; **(B)** group-averaged functional connectivity metrics in MCS; **(C)** 118 functional connectivity that changed significantly between-group differences. L, left; R, right; PreM and SMA, premotor and supplementary motor area; BROCA, Broca’s area; FEF, frontal eye fields; DLPFC, dorsolateral prefrontal cortex; FPA, frontopolar area.

Figures 2A, B show the group-averaged functional connectivity metrics of HC and MCS groups. It can be observed that the averaged correlation coefficient was significantly lower in the MCS group. Among them, 118 connections were still significantly different (*p* < 0.01, FDR correction), and most of these connections were located in the channels of the frontopolar area and DLPFC (Figure 2C).

**FIGURE 3:**
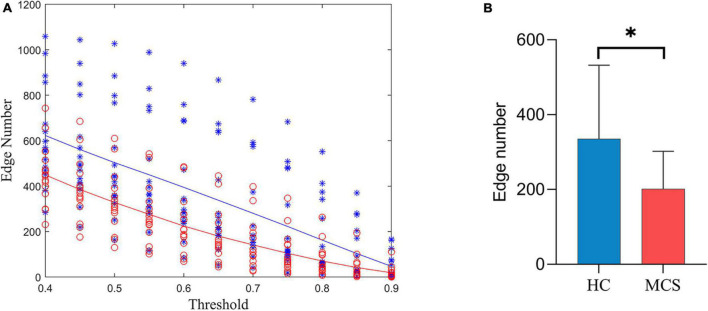
Differences in the number of edges between HC and MCS groups. **(A)** Scatter plot: distribution of edges with different thresholds (0.4–0.9, 0.05 interval). Functional connectivity strength larger than the threshold was defined as edge. HC (blue), MCS (red); **(B)** group differences in the total number of edges. HC, healthy controls; MCS, minimally conscious state. *p < 0.05.

Figure 3A shows the number of edges at different thresholds (0.4–0.9, 0.05 interval) for the HC and MCS groups (Supplementary Tables 1, 2). The average number of edges at different thresholds was taken as each subject’s total number of edges, and the total number of edges in the MCS group was significantly less than in the HC group (Figure 3B).

### 3.3. Between-group differences in network topological properties

**FIGURE 4:**
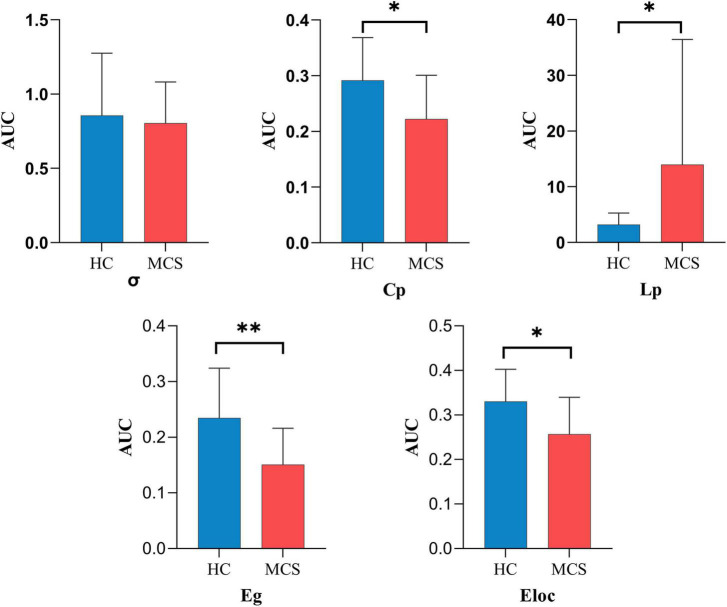
The global topological properties between HC and MCS groups. AUC, area under the curve; σ, small-worldness; Cp, clustering coefficient; Lp, characteristic path length; Eg, global efficiency; Eloc, local efficiency; HC, healthy controls; MCS, minimally conscious state. *p < 0.05, ^**^p < 0.01.

For global topological properties, the AUC of Cp, Eg, and Eloc in the MCS group was significantly lower than in the HC group, and the AUC of Lp in the MCS group was higher than in the HC group. There was no difference in the AUC of σ (Figure 4 and Supplementary Table 3).

For nodal topological properties, the MCS group showed lower NCp and NLe after FDR correction than the HC group. Lower NCp were located in L_FPA (*p* = 0.020), R_DLPFC (*p* = 0.047), R_FEF (*p* = 0.035) (Table 3 and Supplementary Table 4), and lower NLe were located in L_FPA (*p* = 0.020), R_DLPFC (*p* = 0.035) (Table 4 and Supplementary Table 5).

**TABLE 3:** Comparison of NCp between HC and MCS groups.

Regions	NCp		*t*-value	*p*-value (FDR correction)
	HC	MCS		
L_FPA	0.322 ± 0.080	0.225 ± 0.080	3.349	0.020
R_DLPFC	0.288 ± 0.098	0.197 ± 0.097	2.605	0.047
R_FEF	0.319 ± 0.085	0.219 ± 0.107	2.888	0.035

NCp, nodal clustering coefficient; HC, healthy controls; MCS, minimally conscious state; L_FPA, left frontopolar area; R_DLPFC, right dorsolateral prefrontal cortex; R_FEF, right frontal eye field; FDR, false discovery rate.

**TABLE 4:** Comparison of NLe between HC and MCS groups.

Regions	NLe		*t*-value	*p*-value (FDR correction)
	HC	MCS		
L_FPA	0.369 ± 0.071	0.264 ± 0.087	3.677	0.020
R_DLPFC	0.325 ± 0.099	0.224 ± 0.103	2.788	0.035

NLe, nodal local efficiency; HC, healthy controls; MCS, minimally conscious state; L_FPA, left frontopolar area; R_DLPFC, right dorsolateral prefrontal cortex; FDR, false discovery rate.

### 3.4. Correlation between topological properties and CRS-R scale

**FIGURE 5:**
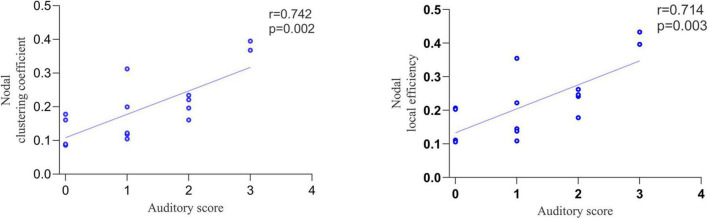
The correlation between nodal topological properties in right dorsolateral prefrontal cortex (DLPFC) and auditory subscale scores.

Correlation analysis was performed between the topological properties with group differences and the CRS-R scale scores of the MCS group. No significant correlation was found between the global topological properties and the total and subscale score of CRS-R. Moreover, we found that two nodal topological properties of right DLPFC, averaged NCp (*r* = 0.742, *p* = 0.002) and NLe (*r* = 0.714, *p* = 0.003), was positively correlated with the auditory subscale scores (Figure 5).

## 4. Discussion

This study explored the topological changes of the functional network in the frontal lobe of MCS patients based on resting-state fNIRS. The study results were as follows: (1) MCS patients had significantly reduced functional connectivity in the frontal lobe, especially in the frontopolar area and DLPFC. (2) MCS patients displayed lower Cp, Eg, Eloc, and higher Lp than the HC group, revealing that frontal network topology changed with the conscious state. (3) NCp and NLe all showed lower in different ROIs, and nodal topological properties in the right DLPFC were correlated with the auditory subscale scores.

### 4.1. Widely disrupted functional connectivity

In this study, we found that resting-state functional connectivity of the whole frontal lobe had severely reduced in MCS patients. Functional connectivity refers to the temporal correlation of neural activity between different brain regions, reflecting the synergistic cooperation of neural activity between brain regions to integrate information (Fingelkurts et al., 2005; Geng et al., 2017). In particular, weakened functional connectivity occurred mainly in the frontopolar area and DLPFC. The frontopolar area and DLPFC are important components of the prefrontal cortex. As the brain’s core, the prefrontal cortex receives and integrates information from many cortical and subcortical areas and participates in conscious perception and cognitive processing (Block, 2020). According to the claim in HOTs theory of consciousness, mental states are conscious by being the target of specific kinds of meta-representation (Seth and Bayne, 2022). Specifically, lower-order representations of auditory signals in posterior cortex would support conscious auditory perception when targeted by the right kind of higher-order meta-representation (especially the prefrontal cortex) (Lau and Rosenthal, 2011; Fleming, 2020). Demertzi et al. also found that functional connectivity was widely declined in DOC patients in the default mode network, frontoparietal network, sensorimotor network, and other resting-state functional networks (Demertzi et al., 2014, 2015). Our study implies a widely disrupted functional connectivity in the frontal lobe of MCS patients, especially in the frontopolar area and DLPFC, further confirming that the changes in the functional connectivity of the frontal lobe may be associated with the altered conscious state (Demertzi et al., 2015; Liu et al., 2017; Moriya and Sakatani, 2018). The functional cooperation of multiple brain regions in the prefrontal cortex played a role in consciousness. Widely disrupted prefrontal functional connectivity means that the prefrontal functional network is synergistically dysfunctional, and the efficiency of conscious processing naturally decreases.

### 4.2. The damage of global topological properties

The results showed that the MCS patients had lower Cp, Eg, Eloc, and higher Lp in the frontal lobe than HC. Cp and Eloc measure the local information transmission capability of the network and represent the functional separation, while Lp and Eg measure the global information transmission capability of the network and represent the functional integration (Bassett and Bullmore, 2017). Weng et al. (2017) found the same changes in Cp and Lp with our results in the whole brain structural network of patients with DOC. Changes in these topological properties in MCS patients not only imply a reduction in global and local information transmission efficiency but also indicate the disturbance in the optimal balance configuration of functional integration and separation in the frontal lobe.

The small-worldness did not show significant differences in MCS and HC groups, which was consistent with previous studies (Tan et al., 2019). The Small-world network can be quantified as shorter Lp and larger Cp. In other words, the Lp and Cp of the small-world network lie between the regular network and the random network (Bassett and Bullmore, 2017). It is found that the brain network of healthy adults satisfies the characteristics of the small-world network, which can consume low-cost neuron resources and efficiently complete information transmission due to the brain function in a dynamic balance between network separation and integration (Bassett and Bullmore, 2006; Samu et al., 2014). This dynamic balance has the inherent ability to support different levels of consciousness and cognitive functions (Wang et al., 2021). A small-world network is an economic network that achieves efficient information transmission with the lowest wiring cost (Samu et al., 2014). Small-worldness is a comprehensive topological property to measure the brain’s functional network. Although there was no significant difference in small-worldness between HC and MCS groups, changes in other network topological properties (Cp, Lp, Eg, and Eloc) indicated that the frontal network configuration of MCS patients tended to have a lower capacity for functional integration and separation. This network state deviates from the optimal critical state, which is not conducive to conscious processing and may consume more neuronal resources. The altered topology caused by pathological injury leads to increased wiring costs and metabolic demands in the frontal lobe. Thus, the frontal network of MCS patients does not belong to the economic network.

### 4.3. Between-group differences in nodal topology properties

We found reduced averaged NCp and averaged NLe of the prefrontal cortex in MCS patients (left frontopolar area and right DLPFC). Previous study had found that the MCS patients showed decreased NCp in the posterior cingulate cortex and right insula cortex and decreased NLe in the right precuneus (Crone et al., 2014). Our findings supplement the existing results and further demonstrate that the activation of the frontoparietal network is related to the conscious state (Crone et al., 2014; Cavinato et al., 2015). One fMRI study showed that higher-level control networks (mainly referring to the frontal and parietal cortex) begin to encode predictive information long before the individual has made a conscious decision (Soon et al., 2008). The prefrontal cortex is also associated with storing conscious decisions and strategy shifts following negative feedback (Soon et al., 2008).

In addition, we found that two nodal topological properties of the right DLPFC, averaged NCp and NLe, were positively correlated with auditory subscale scores. The evaluation of the auditory subscale mainly reflects auditory perception, language comprehension, and executive control of DOC patients (Giacino et al., 2018). The dorsal pathway projects from the primary auditory cortex through the temporal plane across the parietal cortex to the DLPFC, supporting the transmission of auditory perception-motor signals (Hertrich et al., 2021). The DLPFC is involved in auditory information attention, evaluation, and working memory processing (Nakai et al., 2005; Rämä and Courtney, 2005; Chen et al., 2008). It is also a core area of the control network, which is closely related to executive control function (Panikratova et al., 2020). Witt et al. (2010) found that patients with mild traumatic brain injury had significantly reduced activity in the right DLPFC during auditory oddball tasks. NCp and NLe reflect local information transmission capacity. The results of correlation analysis showed that the worse auditory functions, the less efficient local information transmission in the right DLPFC.

## 5. Limitations

Several limitations of this study should be considered. First, the sample size is too small. We should recruit more samples to verify the stability and repeatability of the data. When the sample size is large enough, the MCS group can be subdivided into MCS- and MCS + groups to explore the functional network of different levels of consciousness, which may lead to more accurate results. Secondly, the etiology of consciousness impairment may be a crucial factor in the disturbance of the functional network. We should group different etiology, such as stroke, hypoxic-ischemic encephalopathy (HIE), traumatic brain injury, intracranial surgery, etc. Finally, this study is a non-longitudinal design, and dynamic follow-up is needed in the future to observe whether the correlation results of this study can be further verified with the improvement of patients’ consciousness levels.

## 6. Conclusion

Using resting-state fNIRS to explore the topology of the functional network in the frontal lobe in MCS patients, we found these metrics were widely disrupted in the frontal lobe. Moreover, the functional separation and integration of the frontal network were seriously unbalanced, especially the local information transmission in the prefrontal cortex. We also found that nodal topological properties in the right DLPFC were positively correlated with auditory behavior scores. Our results reveal the disturbance in frontal functional network caused by pathological injury, which may be conducive to understanding the pathological mechanism of MCS patients better.

## Data availability statement

The original contributions presented in this study are included in the article/Supplementary material, further inquiries can be directed to the corresponding authors.

## Ethics statement

This study was conducted in Zhongnan Hospital of Wuhan University, approved by the Medical Research Ethics Committee and Institutional Review Board of Zhongnan Hospital (2021126), and written informed consent was signed by the legal surrogate of each patient. The patients/participants provided their written informed consent to participate in this study.

## Author contributions

HC, GM, YG, and WL: study contributions included study design and conception. GM, YG, SW, JZ, XZ, JL, CH, HH, and TJ: data collection. HC and GM: statistical analysis and interpretation of results. All authors contributed to the article and approved the submitted version.
