# Supplementary material for: Disturbed functional connectivity and topological properties of the frontal lobe in minimally conscious state based on resting-state fNIRS
Source: Front Neurosci. 2023 Feb 10;17:1118395. doi: 10.3389/fnins.2023.1118395 (PMC9950516; doi:10.3389/fnins.2023.1118395)

**Disturbed Functional Connectivity and Topological Properties of the Frontal Lobe in Minimally Conscious State Based on Resting-state fNIRS**

|  | Thresholds | | | | | | | | | | | | mean value | |
| --- | --- | --- | --- | --- | --- | --- | --- | --- | --- | --- | --- | --- | --- | --- |
|  | 0.4 | 0.45 | 0.5 | 0.55 | 0.6 | 0.65 | 0.7 | 0.75 | 0.8 | 0.85 | 0.9 |  | |  |
| HC01 | 470 | 416 | 360 | 311 | 262 | 209 | 152 | 109 | 65 | 31 | 16 | 218.27 | |  |
| HC02 | 857 | 802 | 766 | 733 | 686 | 644 | 584 | 508 | 412 | 274 | 126 | 581.09 | |  |
| HC03 | 1059 | 1044 | 1027 | 989 | 940 | 867 | 782 | 683 | 552 | 370 | 167 | 770.91 | |  |
| HC04 | 569 | 490 | 419 | 365 | 296 | 242 | 181 | 116 | 67 | 28 | 8 | 252.82 | |  |
| HC05 | 597 | 529 | 441 | 361 | 282 | 217 | 153 | 101 | 61 | 28 | 8 | 252.55 | |  |
| HC06 | 984 | 940 | 885 | 829 | 759 | 674 | 592 | 482 | 342 | 204 | 71 | 614.73 | |  |
| HC07 | 484 | 411 | 352 | 288 | 242 | 190 | 140 | 98 | 62 | 25 | 12 | 209.45 | |  |
| HC08 | 554 | 497 | 446 | 396 | 344 | 279 | 225 | 170 | 134 | 93 | 54 | 290.18 | |  |
| HC09 | 558 | 471 | 402 | 335 | 284 | 219 | 164 | 112 | 61 | 22 | 7 | 239.55 | |  |
| HC10 | 640 | 568 | 493 | 420 | 338 | 262 | 183 | 123 | 56 | 29 | 6 | 283.45 | |  |
| HC11 | 517 | 433 | 358 | 307 | 245 | 181 | 128 | 89 | 46 | 30 | 14 | 213.45 | |  |
| HC12 | 885 | 849 | 798 | 750 | 690 | 637 | 574 | 478 | 374 | 280 | 162 | 588.82 | |  |
| HC13 | 382 | 307 | 251 | 197 | 155 | 121 | 89 | 72 | 47 | 31 | 8 | 150.91 | |  |
| HC14 | 284 | 219 | 163 | 117 | 84 | 52 | 40 | 18 | 10 | 5 | 0 | 90.18 | |  |
| HC15 | 452 | 387 | 324 | 280 | 236 | 184 | 128 | 85 | 54 | 32 | 15 | 197.91 | |  |
| HC16 | 673 | 617 | 569 | 520 | 472 | 427 | 374 | 317 | 257 | 171 | 76 | 406.64 | |  |

Table. S1 The edge number at different thresholds (0.4-0.9, 0.05 interval) in HC.

Table. S2 The edges number at different thresholds (0.4-0.9, 0.05 interval) in MCS group

|  | Thresholds | | | | | | | | | | | | mean value | |
| --- | --- | --- | --- | --- | --- | --- | --- | --- | --- | --- | --- | --- | --- | --- |
|  | 0.4 | 0.45 | 0.5 | 0.55 | 0.6 | 0.65 | 0.7 | 0.75 | 0.8 | 0.85 | 0.9 |  | |  |
| MCS01 | 297 | 219 | 166 | 114 | 80 | 54 | 31 | 21 | 13 | 9 | 3 | 91.55 | |  |
| MCS02 | 231 | 176 | 130 | 102 | 69 | 45 | 27 | 15 | 8 | 2 | 1 | 73.27 | |  |
| MCS03 | 553 | 497 | 440 | 391 | 332 | 274 | 219 | 166 | 101 | 58 | 19 | 277.27 | |  |
| MCS04 | 656 | 605 | 564 | 526 | 485 | 445 | 398 | 347 | 263 | 194 | 111 | 417.64 | |  |
| MCS05 | 743 | 685 | 610 | 542 | 481 | 410 | 336 | 248 | 178 | 100 | 43 | 397.82 | |  |
| MCS06 | 426 | 355 | 304 | 242 | 186 | 144 | 110 | 69 | 42 | 18 | 6 | 172.91 | |  |
| MCS07 | 472 | 401 | 354 | 299 | 253 | 203 | 171 | 141 | 97 | 59 | 20 | 224.55 | |  |
| MCS08 | 450 | 371 | 310 | 250 | 187 | 139 | 93 | 55 | 32 | 12 | 7 | 173.27 | |  |
| MCS09 | 446 | 382 | 321 | 268 | 208 | 166 | 123 | 88 | 60 | 37 | 18 | 192.45 | |  |
| MCS10 | 514 | 457 | 391 | 332 | 276 | 238 | 194 | 144 | 108 | 65 | 30 | 249.91 | |  |
| MCS11 | 367 | 311 | 246 | 197 | 149 | 105 | 70 | 44 | 27 | 11 | 4 | 139.18 | |  |
| MCS12 | 299 | 233 | 174 | 133 | 96 | 65 | 50 | 27 | 13 | 6 | 0 | 99.64 | |  |
| MCS13 | 399 | 342 | 283 | 230 | 179 | 127 | 93 | 60 | 39 | 22 | 11 | 162.27 | |  |
| MCS14 | 418 | 349 | 293 | 233 | 177 | 135 | 97 | 66 | 30 | 11 | 3 | 164.73 | |  |
| MCS15 | 466 | 394 | 337 | 281 | 210 | 154 | 110 | 76 | 46 | 23 | 7 | 191.27 | |  |

Table. S3 The AUC of global topological properties in HC and MCS group

| aSigma (σ） | | aCp | | aLp | | aEg | | aEloc | |
| --- | --- | --- | --- | --- | --- | --- | --- | --- | --- |
| HC | MCS | HC | MCS | HC | MCS | HC | MCS | HC | MCS |
| 1.2708 | - | 0.2575 | 0.1238 | 6.8506 | 22.8500 | 0.1521 | 0.0963 | 0.2987 | 0.1525 |
| 0.5343 | - | 0.3849 | 0.0833 | 0.7713 | 88.4632 | 0.3494 | 0.0495 | 0.4160 | 0.0963 |
| 0.5217 | 0.5543 | 0.4249 | 0.2679 | 0.6552 | 2.2782 | 0.4065 | 0.1975 | 0.4497 | 0.3055 |
| 2.0301 | 0.4577 | 0.2462 | 0.3641 | 5.0753 | 1.0779 | 0.2107 | 0.2722 | 0.2965 | 0.3924 |
| 0.9299 | 0.5224 | 0.2794 | 0.3408 | 3.4504 | 1.1528 | 0.2339 | 0.2879 | 0.3330 | 0.3783 |
| 0.5107 | - | 0.3828 | 0.1901 | 0.7866 | 14.8437 | 0.3646 | 0.1087 | 0.4146 | 0.2212 |
| 0.6979 | 0.9095 | 0.2414 | 0.2983 | 3.8587 | 2.3159 | 0.1772 | 0.1809 | 0.2800 | 0.3427 |
| 0.6921 | - | 0.2824 | 0.1974 | 1.6768 | 13.4960 | 0.2191 | 0.1296 | 0.3280 | 0.2387 |
| 0.7745 | 0.7005 | 0.2496 | 0.2546 | 4.6505 | 4.5505 | 0.1961 | 0.1425 | 0.2918 | 0.2908 |
| - | 0.9107 | 0.2763 | 0.2799 | 4.4871 | 2.6489 | 0.2141 | 0.1614 | 0.3207 | 0.3190 |
| 1.1183 | 1.1547 | 0.2443 | 0.1622 | 4.0017 | 15.4754 | 0.1984 | 0.0855 | 0.2946 | 0.1866 |
| 0.4906 | - | 0.3985 | 0.1575 | 0.7974 | - | 0.3488 | 0.1069 | 0.4228 | 0.1923 |
| 0.8521 | 0.7861 | 0.2587 | 0.1835 | 5.6164 | 6.3518 | 0.1352 | 0.1230 | 0.2941 | 0.2175 |
| - | 1.2457 | 0.1286 | 0.2104 | - | 13.8989 | 0.0973 | 0.1490 | 0.1589 | 0.2526 |
| 1.0284 | - | 0.2584 | 0.2213 | 4.4970 | 6.3615 | 0.1712 | 0.1741 | 0.2985 | 0.2651 |
| 0.5195 | - | 0.3507 | - | 1.0832 | - | 0.2755 | - | 0.3842 | - |

Table. S4 The AUC of nodal clustering coefficient in HC and MCS group

| L_FPA | | R_DLPFC | | R_FEF | |
| --- | --- | --- | --- | --- | --- |
| HC | MCS | HC | MCS | HC | MCS |
| 0.21323 | 0.081342 | 0.17743 | 0.11822 | 0.34297 | 0.057381 |
| 0.39269 | 0.12929 | 0.43694 | 0.10472 | 0.43059 | 0.1152 |
| 0.4381 | 0.21171 | 0.43815 | 0.23426 | 0.4276 | 0.17092 |
| 0.27309 | 0.36304 | 0.27219 | 0.3681 | 0.25959 | 0.40438 |
| 0.24139 | 0.36968 | 0.2752 | 0.39524 | 0.34323 | 0.37882 |
| 0.38719 | 0.23978 | 0.41149 | 0.16118 | 0.42239 | 0.21887 |
| 0.32645 | 0.30833 | 0.26072 | 0.31281 | 0.19232 | 0.2964 |
| 0.35381 | 0.17525 | 0.28193 | 0.19652 | 0.31556 | 0.19998 |
| 0.29599 | 0.22593 | 0.23114 | 0.22096 | 0.26726 | 0.29012 |
| 0.30936 | 0.26502 | 0.29834 | 0.17825 | 0.38897 | 0.30836 |
| 0.29546 | 0.21246 | 0.24887 | 0.19974 | 0.28626 | 0.10463 |
| 0.43078 | 0.154 | 0.4315 | 0.085913 | 0.30382 | 0.14609 |
| 0.28878 | 0.24619 | 0.24529 | 0.12236 | 0.28223 | 0.25922 |
| 0.15679 | 0.16395 | 0.10181 | 0.16116 | 0.13691 | 0.076118 |
| 0.31926 | 0.22824 | 0.19297 | 0.08943 | 0.29769 | 0.26424 |
| 0.42552 | - | 0.30214 | - | 0.41347 | - |

Table. S5 The AUC of nodal local efficiency in HC and MCS group

| L_FPA | | R_DLPFC | |
| --- | --- | --- | --- |
| HC | MCS | HC | MCS |
| 0.26957 | 0.10789 | 0.20885 | 0.14503 |
| 0.43166 | 0.15137 | 0.46791 | 0.10912 |
| 0.46233 | 0.24881 | 0.4638 | 0.26217 |
| 0.33453 | 0.40543 | 0.32923 | 0.39653 |
| 0.29036 | 0.41671 | 0.31856 | 0.43321 |
| 0.4109 | 0.27988 | 0.45022 | 0.17822 |
| 0.37692 | 0.35373 | 0.29049 | 0.3548 |
| 0.39064 | 0.21814 | 0.33171 | 0.2414 |
| 0.35296 | 0.26357 | 0.26692 | 0.24628 |
| 0.35586 | 0.31616 | 0.35133 | 0.20668 |
| 0.37247 | 0.23863 | 0.30488 | 0.22215 |
| 0.45838 | 0.18973 | 0.45781 | 0.11158 |
| 0.33143 | 0.29681 | 0.28221 | 0.13831 |
| 0.20974 | 0.2023 | 0.12439 | 0.20325 |
| 0.38667 | 0.27193 | 0.21524 | 0.10597 |
| 0.46192 | - | 0.33331 | - |

Abbreviations: HC, healthy controls; MCS, minimally conscious state; AUC, area under the curve; σ, small-worldness; Cp, clustering coefficient; Lp, characteristic path length; Eg, global efficiency; Eloc, local efficiency; NCp, nodal clustering coefficient; NLe, nodal local efficiency; L_FPA, left frontopolar area; R_DLPFC, right dorsolateral prefrontal cortex; R_FEF, right frontal eye field.

Figure S1 The distribution of 50 channels after the three midline channels are removed. Including the PreM and SMA (red, Premotor and supplementary motor area), FEF (purple, frontal eye fields), BROCA (green, Broca's area), FPA (yellow, frontopolar area), and DLPFC (blue, dorsolateral prefrontal cortex).


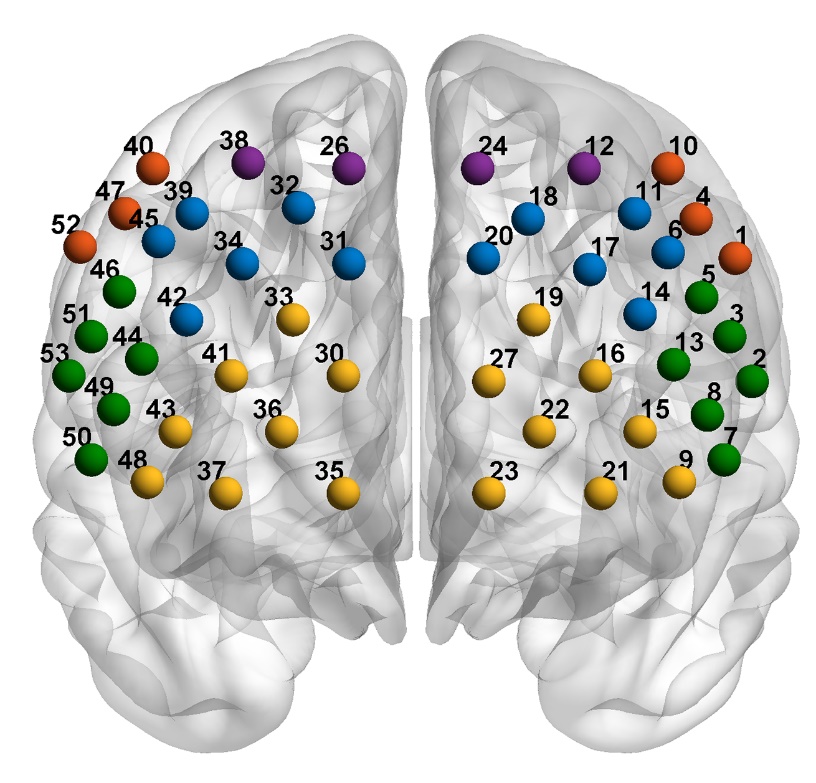

Supplement: Supplementary file 1 [file Data_Sheet_1.docx]
